# A method for time‐independent film dosimetry: Can we obtain accurate patient‐specific QA results at any time postirradiation?

**DOI:** 10.1002/acm2.13534

**Published:** 2022-01-20

**Authors:** Leon Dunn, Guy Godwin, James Hellyer, Xiaolei Xu

**Affiliations:** ^1^ St Vincent's GenesisCare Centre for radiation oncology St Vincent's Hospital Basement Level Building C, 41 Victoria Parade, Fitzroy VIC 3065 Melbourne Victoria 3065 Australia; ^2^ Redland Icon Cancer Care Bayside Business Park, 16/24 Weippin St, Cleveland QLD Brisbane Queensland 4163 Australia; ^3^ Macquarie University GenesisCare Centre for radiation oncology Hospital Building Suite 1, Level B2, 3 Technology Pl, Macquarie University NSW Sydney New South Wales 2109 Australia

## Abstract

**Aim:**

The aim of this work was twofold. (1) To investigate and present a comparison between EBT3 and EBT‐XD in terms of postirradiation color changes. (2) Create an automated workflow to allow radiochromic film (EBT3/XD) to be scanned and converted to dose accurately at any postirradiation time.

**Materials and Methods:**

Ten GafChromic EBT‐XD calibration films were exposed in 2 Gy increments up to 18 Gy. Calibrates were then scanned at 5‐min intervals postirradiation over 24 h using an AutoHotKey script, resulting in 288 TIFF images. Following the 24‐h scanning period, a MATLAB script was used to automatically read the tiff images and create a series of 288 calibration curves distinct in time which is termed as the “Temporal Calibration Model” (TCM). The model is saved as a series of polynomial fit coefficients to net optical density as a function of dose, timestamped in 5‐min increments. Ten patient‐specific film measurements (5 × EBT‐XD and 5 × EBT3) were then carried out and scanned using the same 5‐min scan intervals from 5 min postirradiation to 24 h postirradiation. The TCM was then automatically applied using eFilmQA software to convert the patient‐specific QA films to dose by applying the relevant calibration curve from the TCM, corresponding to the arbitrary postirradiation time that the film was scanned. Each dose plane at postirradiation scan intervals of 5 min up to 20 h was then compared to the ground‐truth dose plane using gamma analysis.

**Results:**

Gamma pass rates using the TCM at time *t*, normalized to the pass rate after 20 h postirradiation, were found to have a maximum coefficient of variation of 3% over any postirradiation time. Conversely, not using the TCM resulted in coefficients of variation of up to 39%. Clinical implementation of this method showed an average accuracy of 2.8% when comparing the clinical result to the TCM result.

**Conclusions:**

We have developed a methodology that allows radiochromic film to be accurately used as a dosimeter at any arbitrary scan postirradiation time, whereas previously, waiting periods of 16–24 h before readout were needed to ensure the postirradiation changes had stabilized. The creation of a TCM can enable results from radiochromic film measurements to be obtained quickly postirradiation. Using a conventional single calibration curve generated at 20 h postirradiation can result in gamma pass‐rate difference of up to 75% for measurement films scanned at a much shorter postirradiation time.

## 1 INTRODUCTION

Radiochromic film consists of a single or double layer of radiation‐sensitive organic microcrystal monomers deposited on a thin polyester base or sandwiched between polyester layers with transparent coatings.^1^ As of 2011, radiochromic film for use in radiotherapy applications has been primarily sold commercially by Ashland (Ashland Advanced Materials, Bridgewater, NJ) as GafChromic. Both EBT3 and EBT‐XD are products that have been well characterized ^2^, ^3^, ^4^, ^5^, ^6^, ^7^, ^8^, ^9^, ^10^, ^11^, ^12^ and are established dosimeters for use in patient‐specific QA of Intensity‐Modulated Radiation Therapy (IMRT),^2^, ^3^, ^13^ Volumetric Modulated Arc Therapy (VMAT),^14^ and Stereotactic Radiotherapy (SABR/SRT)^7^, ^15^ plans. The superior spatial resolution of radiochromic film makes it particularly useful for SABR verification, which is characterized by small fields and high dose gradients.

With proper handling and processing protocols, radiochromic film is an accurate and reproducible dosimeter to within 5%.^16^, ^17^, ^18^ However, a well‐known limitation of using radiochromic film is the need to wait several hours for the postirradiation changes in optical transmittance of an irradiated film to decrease over time ^19^ thereby reducing the measurement uncertainty.^20^ These changes to optical density (OD) with postirradiation time are proportional to the logarithm of postirradiation time.^18^, ^21^ It has been shown in several studies ^2^, ^14^, ^20^, ^21^, ^22^, ^23^, ^24^, ^25^, ^26^, ^27^, ^28^ that the postirradiation OD increases with time whereas the rate of OD change of an irradiated film decreases with time. This effect postirradiation is due to ionizing radiation creating an evolving solid‐state polymerization in crystals within the active layer. As time goes on, the rate of polymerization decreases, which causes the chemical changes to stabilise in a linear manner with log_10_ (time).^20^, ^29^ As a result of this continual polymerization with time, errors can occur if a given dose‐response calibration is established by scanning calibration films at a time, *t*, postirradiation and then applying this dose‐response curve for measurement of films scanned at a different postirradiation times, Δ*t*. This error is reduced by minimizing the ratio of *t/*Δ*t*, that is, by scanning the measurement films at a set time‐postirradiation which corresponds to the same time‐postirradiation at which the dose‐response calibration was determined.^20^ Published literature suggests that a postirradiation period of 16–24 h is suitable to minimize this uncertainty.^20^, ^28^, ^30^

Previous work has stated that this effect can be mitigated by applying relative correction factors that are correct for the difference between the time of irradiation and scanning for calibration and measurement films, respectively. These methods involve exposing one or more “reference” or known‐dose films immediately after the measurement and scanning those films along with the measurement film. A method that is used commercially was put forward by Lewis et al. ^29^ which states that postirradiation effects can be corrected using this type of scaling method. Using this method, net values for all measured doses at time‐postirradiation (Δ*t*) can be scaled by the ratio of the net value of the reference dose at a time, *t* (calibration time) after irradiation divided by the net value of the reference dose Δ*t* minutes after irradiation.^29^ The minimum postirradiation time that can be used in this method is suggested to be 4× the difference in time between the measurement film irradiation and the reference dose irradiation to reduce the error to <0.5%.^29^ For example, if the reference dose film was irradiated 5 min after the measurement dose film, then the user would wait 20 min and read both films out in one scan.

The recent AAPM Task Group Report 235 ^20^ recommends the “one‐scan” protocol with embedded recalibration methods to compensate for interscan variability, changes in film response due to varying environmental conditions, and postirradiation OD growth of radiochromic films. The work presented herein explicitly deals with the postirradiation growth of OD and the application of different approaches to account for postirradiation growth. It is important to note that the methods presented herein can be used in conjunction with the one‐scan protocol, combining all the benefits it awards in terms of minimizing interscan variability, changes in film response due to varying environmental conditions, and postirradiation OD growth.

The “one‐scan” protocol is demonstrated to work ^29^ but it has limitations in terms of accounting for postirradiation growth. It assumes that for every pixel with arbitrary dose, *D(i,j)* at time Δ*t* postirradiation, the correction for the postirradiation behavior is the same, that is, a simple relative or constant linear correction based on two or more points no matter the arbitrary dose value. We will show that this is approximately true after equilibrium which is attained for all dose levels in the matrix. However, as the time‐to‐scan (Δ*t*) postirradiation is reduced, accounting for this difference in the postirradiation response, which is both a function of time *and* dose/OD, becomes nontrivial. Ideally, what is needed for accurate dosimetry as soon as possible after irradiation is a full dose‐response curve over the complete range of doses at every point in postirradiation time. We propose that this temporal dose‐response correction, or Temporal Calibration Model (TCM), accurately enables the full range of measured OD values in the matrix at any time *t* postirradiation to be converted to dose.

With the use of SABR and SRT expanding rapidly ^31^ not just in terms of patient numbers, but also in the range of anatomical treatment sites, the delay between irradiation and scanning time when processing radiochromic film is a well‐known bottleneck in the treatment chain. Given that a patient's time‐to‐treatment is continuing to decrease with the implementation of automated planning and other workflow efficiency gains being implemented, it would be considered advantageous to have results of patient‐specific QA sooner, rather than later. Further, it is generally considered better to know the results of patient‐specific QA as soon as possible so that replanning or errors in dose delivery can be corrected. In this work, an automated methodology is presented and validated that allows radiochromic film to be read‐out and accurately processed as soon as needed at any reasonable postirradiation time.

## 2 MATERIALS AND METHODS

### 2.1 Film calibration and scanning methodology

Ten EBT‐XD (#04282002) calibrates with size 4 × 3 cm^2^ were exposed to doses from 2 to 18 Gy, in 2 Gy increments, on an Elekta VersaHD linear accelerator under reference conditions of SSD = 90 cm, depth = 10 cm, with a field‐size of 10 × 10 cm^2^ using a 6 MV flattening filter‐free (FFF) beam in a 30 × 30 × 30 cm^3^ solid water stack.

Following irradiation, the ten films were arranged on an Epson Expression 11000XL Photo scanner (Seiko Epson Corporation, Nagano, Japan) at the center of the scan plane in two rows of five with consistent orientation. Scanner settings for version 3.222 of the EPSON Scan software were then set to produce 48‐bit color, 72 DPI images with no color correction. At exactly 5 min after the final calibration film irradiation was finished, an AutoHotKey (AutoHotkey Foundation LLC) script was used to simulate a spacebar keypress event on the scan button every 5 min (300 000 ms). AutoHotkey is a free, open‐source scripting language for Windows that allows users to easily create scripts for tasks such as form filling, autoclicking, macros, etc.

After 24 h, the “insatiable birdy” script will produce 288 calibration film‐set TIFF images that have been acquired at 5‐min intervals. It should be noted that 5‐min intervals were chosen for this work, however, any arbitrary interval between keypress events can be set, with the obvious consideration of the time it takes to scan the film. This method was then repeated for a set of calibration films of EBT3 (#03122004).

**FIGURE 1:**
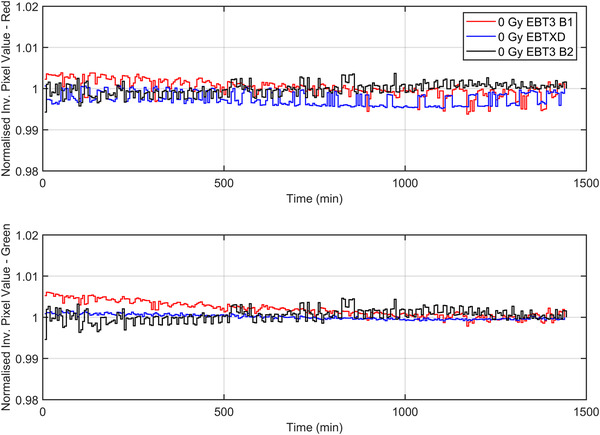
Mean pixel values of an unexposed film as a function of time normalized to mean pixel value at 24 h postirradiation. The red and green channel values for 2 × EBT3 films from separate batches and an EBT‐XD batch are shown.

To examine the effects of repeat scanning over 24 h at 5‐min intervals, the unexposed film of the set was used to determine whether any scanner/bulb warming effects could have an impact when using this method. To quantify this, the mean pixel value for the red and green channels at each scan point in time for the unexposed film was normalized to the mean pixel value at 24 h. The results of this test are shown in Figure 1.

### 2.2 Automated creation of calibration curves and TCM

MATLAB (The MathWorks, Natick, Massachusetts, United States) based software called eFilmQA (IsoAnalytics Pty Ltd, Melbourne, Australia) was used to automatically read the 288 calibration set images, detect each individual calibrate, and assign a consistent region‐of‐interest (ROI) at the center of each, with an area equal to one‐third the area of the calibrate. The mean OD of each ROI was then calculated according to Equation 1:

(1) $$OD_{net}=\log_{10}\left(\frac{I_{0}}{I}\right),$$

where *I_0_* is the mean pixel value of the zero‐dose ROI for the red or green channel and *I* is the mean pixel value for each nonzero calibrated ROI. Third order polynomial curves were then fitted to the values of *OD_net_* against dose [Gy] resulting in 288 separate calibration curves with ten dose points each. The coefficients (*a, b, c, d*) for the third order polynomial fits are then saved in a comma‐separated values (.csv) file with the data format [time, a, b, c, d] along with the mean ROI *OD_net_* for the background zero‐dose films. It is this .csv file that is the TCM for the batch of film, that is, a series of calibration curves distinct in postirradiation time. For this work, the mean OD_net_ for each calibrate was also recorded to analyze the changes over time.

### 2.3 Retrospective patient‐specific QA validation of the TCM

Five EBT3 (5 × #03122004) and EBT‐XD (5 × #04282002) patient‐specific QA cases were delivered on an Elekta VersaHD linear accelerator utilizing 6 MV FFF photon beams. The treatment sites and prescriptions for these are shown in Table 1. Note that the table lists the dose in Gy per #, however, there is a range of doses from 0 Gy to over and above these doses given the nature of stereotactic treatment plans often being prescribed to a lower isodose line.

**TABLE 1:** Patient‐specific QA patient measurement cases and corresponding dose per fraction

Patient #	Treatment site	Dose/# (Gy)
EBT‐XD‐1	Brain	8
EBT‐XD‐2	Lung	12
EBT‐XD‐3	Brain	8
EBT‐XD‐4	R‐Ilium	12
EBT‐XD‐5	Spine	12
EBT3‐1	Lung	12
EBT3‐2	Spine	10
EBT3‐3	Sacrum	8
EBT3‐4	Brain	10
EBT3‐5	Brain	8

Films were exposed in either a custom Perspex cylindrical phantom for SBRT cases or the CIRS Multilesion Brain QA Phantom model 037 for intracranial cases. Following irradiation, the same readout process utilizing the AutoHotKey script detailed in 0 was performed for each measurement film. At the end of the 24‐h automated scanning process, 288 images per measurement film scanned at 5‐min intervals postirradiation are produced.

The validation of the method was performed by comparing the gamma pass rates at 1%/1 mm dose‐difference/distance to agreement when using the model, to the gamma passrates without the model at varying time points postirradiation. eFilmQA was used for all gamma analysis and the TCM is applied in the software by asking the user to input the postirradiation time that the scan corresponds to. This time value is then used as a lookup value to match the postirradiation time in the TCM and apply those calibration coefficients to convert from OD_net_ to dose. For all ten patients, the film dose‐plane array at 20 h postirradiation was taken as the ground‐truth image, with all gamma pass rates at different times normalized to the pass rate at 20 h postirradiation.

### 2.4 Clinical patient‐specific QA implementation and initial results

Six patient‐specific QA cases were used in the implementation trial across two separate facilities and private radiation oncology providers. Four cases at Organization A were intracranial, consisting of multiple planning target volumes (PTVs) planned with Eclipse (Varian Medical Systems, Palo Alto, California) and delivered on a Varian TrueBeam linear accelerator using EBT‐XD localized in a StereoPHAN (Sun Nuclear, Melbourne, Florida, USA) phantom. Two additional cases, irradiated at Organization B, consisted of two SRT cases (left sinus and right temporal lobe). The two cases at Organization B were planned by Monaco (Elekta AB, Stockholm, Sweden) and irradiated in a CIRS multilesion brain QA phantom, model 037 (CIRS, Norfolk, Virginia, USA), using an Elekta Versa HD linear accelerator.

Each facility derived its own TCM and applied it to these patient cases using eFilmQA. Organisation A uses patient‐specific QA criteria of 5%/2 mm while Organisation B uses 5%/1.5 mm. Because these criteria are somewhat insensitive, each site's results were reprocessed at 2%/1 mm at arbitrary scan times postirradiation. The results obtained using the TCM were then compared to the results obtained using a standard calibration curve at those time points.

## 3 RESULTS

### 3.1 Scanner stability with repeat scanning over 24 h

Figure 1 shows the mean normalized inverse pixel value of an unexposed film scanned at 5‐min intervals over 24 h as part of the calibration set. The reproducibility of the scanner was within 1% over the 24‐h scanning period when normalized to the mean inverse pixel value after 24 h of scanning. These data suggest that for this Epson Expression 11000XL scanner, the stability is such that warming/scanner reproducibility effects do not need to be explicitly considered when generating the TCM. Furthermore, the unexposed calibrate is used in the generation of each of the calibration curves of the TCM and, hence, the scanner response is factored into the calibration curve generated at that time point.

### 3.2 Postirradiation OD_net_ growth as a function of dose and time

**FIGURE 2:**
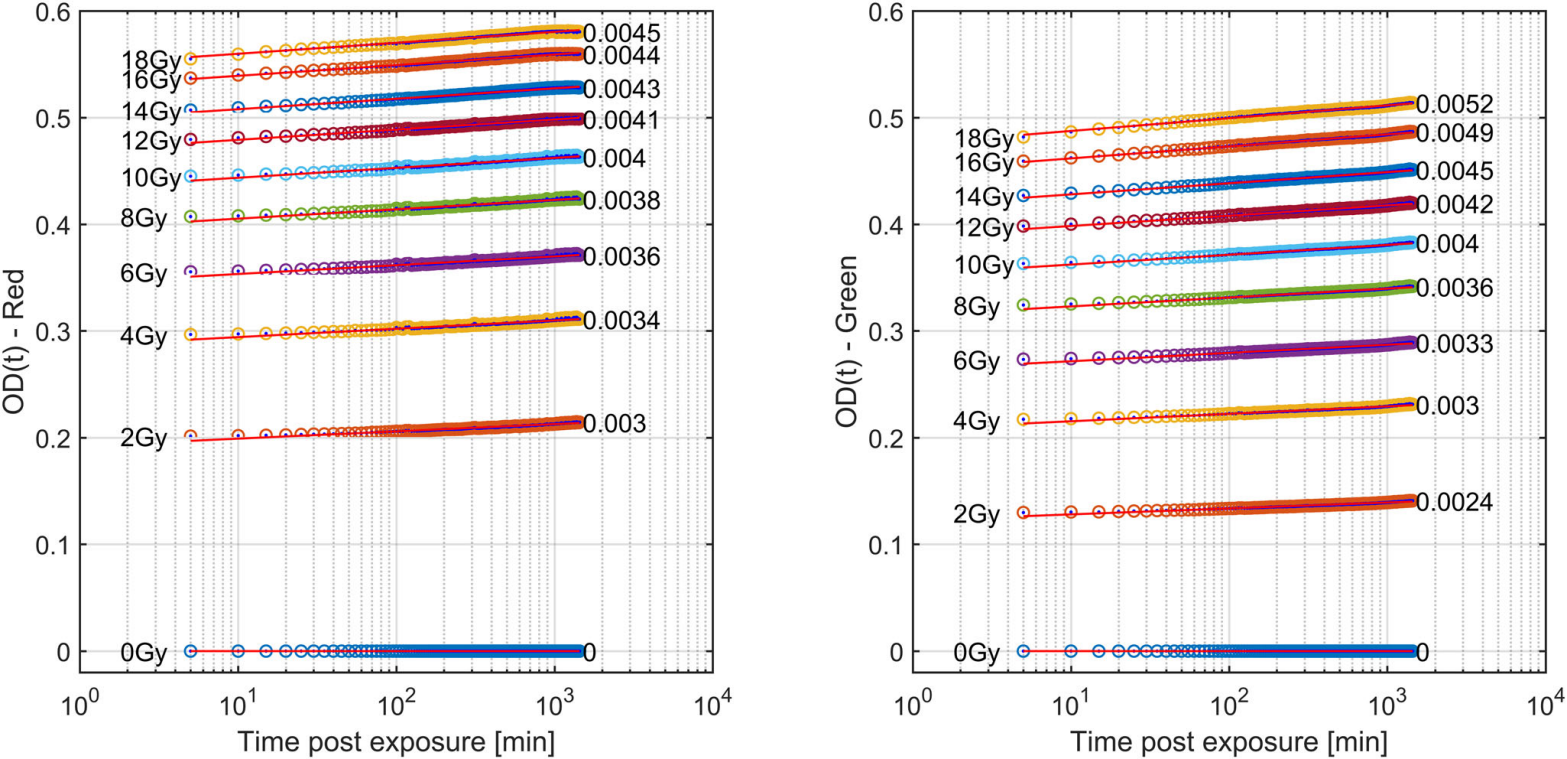
EBT3 batch #03122004 OD_net_ as a function of log time for a period of 24 h postirradiation for doses ranging from 0 to 18 Gy. The red channel is shown on the left with the green on the right. The gradient of a linear line of best fit to the data is shown on the right‐hand side of each series. The temporal resolution of these data is 5 min.

Figure 2 shows the OD_net_ growth for each dose level as a function of log_10_ postirradiation time for EBT3 batch #03122004. The value on the left‐hand side of each series corresponds to the dose level and the value on the right‐hand side of each series shows a gradient of a linear line of best fit. The different gradients per dose level show that the postirradiation OD_net_ changes are a function of irradiated dose level, color channel, and postirradiation time. Both the red and green channels show a consistent increase in the rate of color change with increasing dose.

**FIGURE 3:**
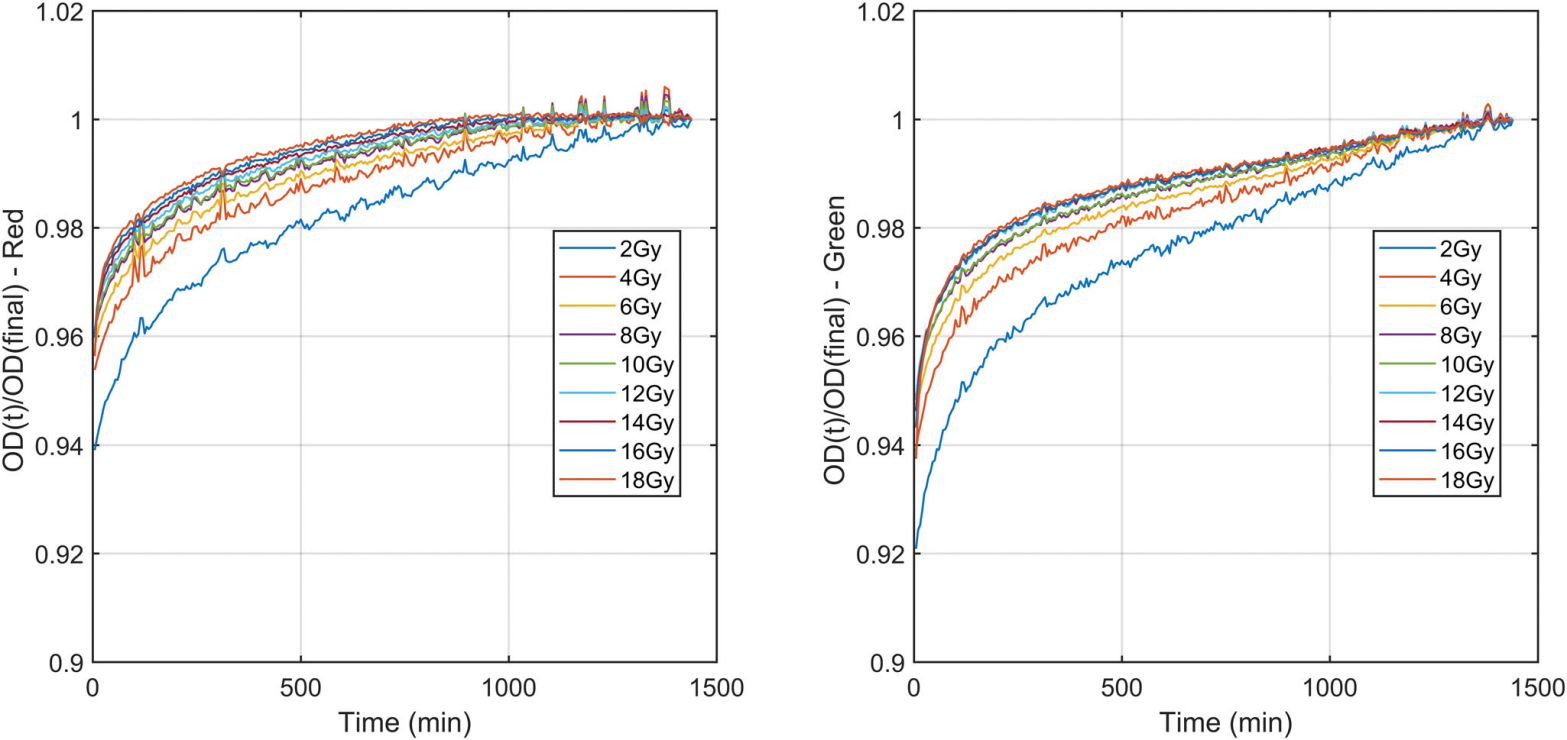
EBT3 batch #03122004 normalized OD_net_ as a function of time for a period of 24 h postirradiation for doses ranging from 0 to 18 Gy. The red channel is shown on the left with the green on the right. Each Net OD at time *t* is normalized to the Net OD at 24 h postirradiation. Note the y‐axis starts at 0.9 to help visualize the difference between the datasets. The temporal resolution of these data is 5 min

Figure 3 shows the normalized OD_net_ at time *t* normalized to the final OD_net_ at 24 h postirradiation for a batch of EBT3. The overall changes in OD_net_ are dependent on dose level and color channel. Here, the overall change reported is the percentage increase in the normalized OD_net_ between the first and final scan. For EBT3 shown here, the 2 Gy dose‐level calibrate shows an overall change of 6.1%, with the 18 Gy film changing by 4.4% for the red channel. For the green channel, the magnitude of these changes over time increases to 7.9% and 6.3% for the 2 and 18 Gy films, respectively.

**FIGURE 4:**
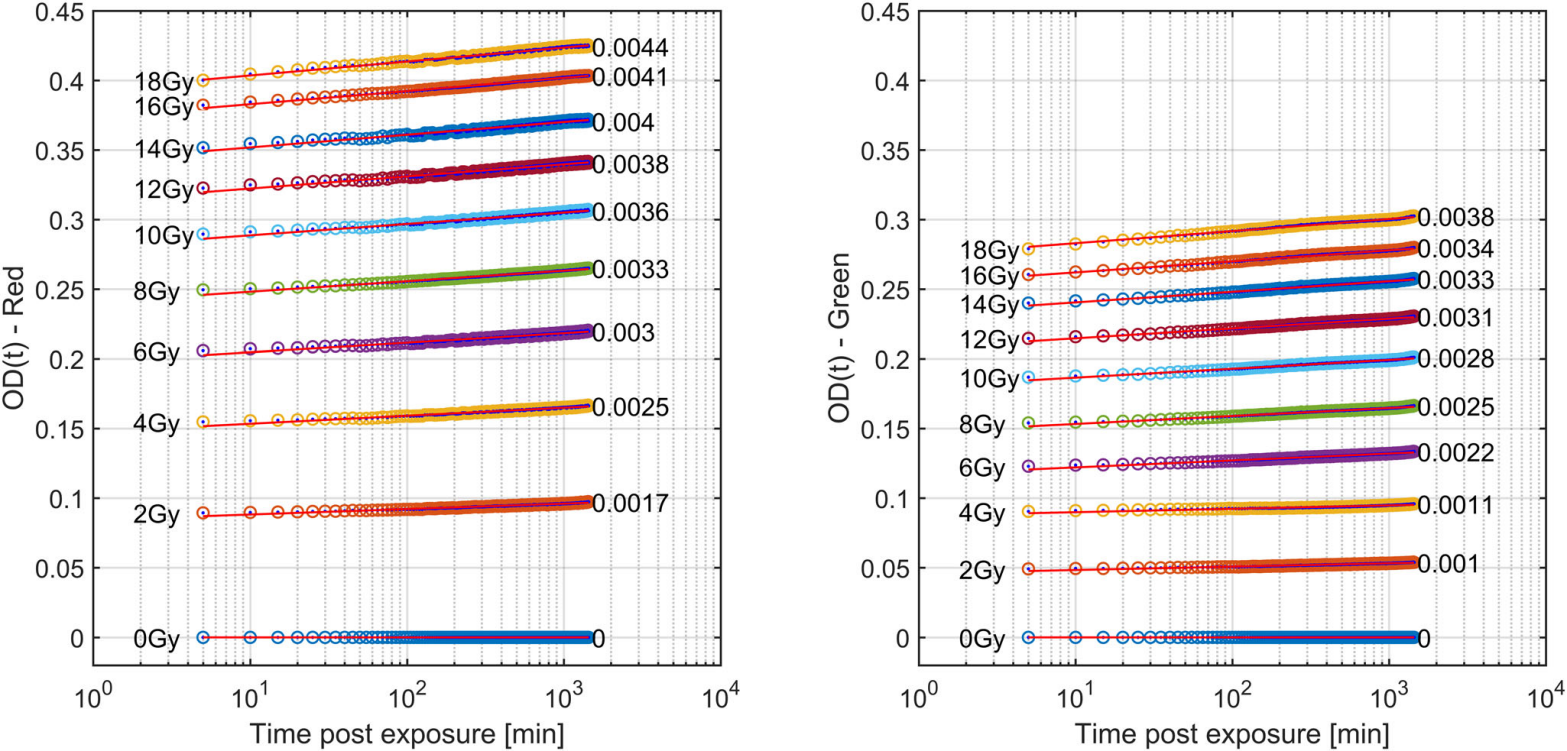
EBT‐XD batch #04282002 OD_net_ as a function of log time for a period of 24 h postirradiation for doses ranging from 0 to 18 Gy. The red channel is shown on the left with the green on the right. The gradient of a linear line of best fit to the data is shown on the right‐hand side of each dataset. The temporal resolution of these data is 5 min

**FIGURE 5:**
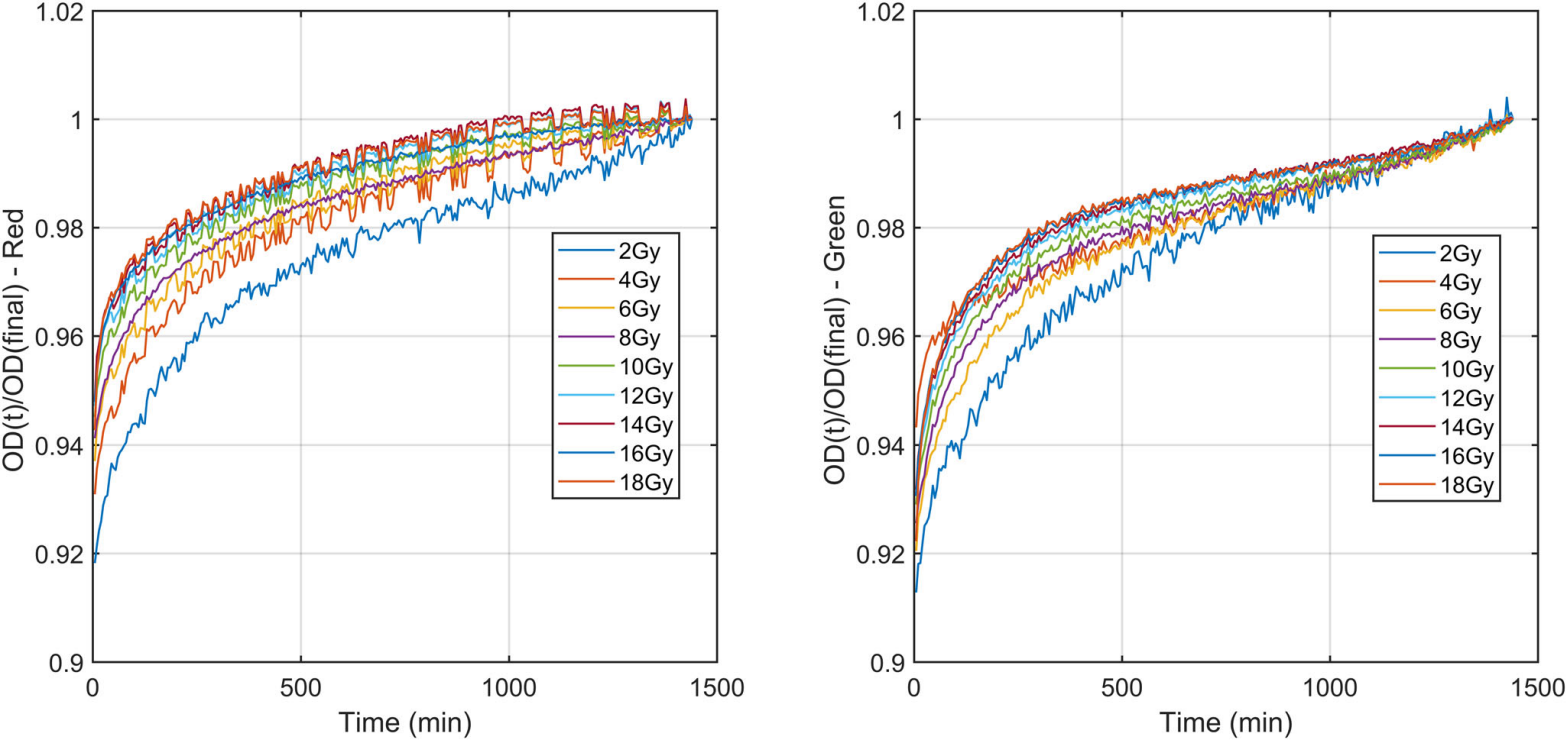
EBT‐XD batch #04282002 normalized OD_net_ as a function of time for a period of 24 h postirradiation for doses ranging from 0 to 18 Gy. The red channel is shown on the left with the green on the right. Each Net OD at time *t* is normalized to the Net OD at 24 h postirradiation. Note the y‐axis starts at 0.9 to help visualize the difference between the datasets. The temporal resolution of these data is 5 min

Figures 4 and 5 show the same data but for the batch of EBT‐XD. Compared with EBT3, greater magnitudes of change over 24 h for all dose levels with the same inverse relationship between the magnitude of postirradiation color change and dose are seen. Here, the 2 and 18 Gy films have 8.2 and 5.7% change in OD_net_ over 24 h postirradiation for the red channel, increasing to 8.7 and 7.8% for the green channel, respectively.

### 3.3 Retrospective patient‐specific QA validation of the TCM

**FIGURE 6:**
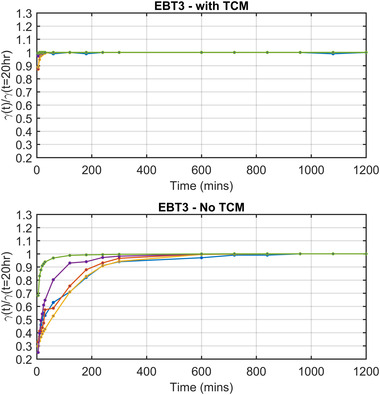
EBT3 normalized gamma pass rates at 1%/1 mm at each time point postirradiation using a TCM (top) and using a single calibration curve generated at 20 h postirradiation (bottom). Each gamma pass‐rate score is normalized to the gamma pass rate of the film at 20 h.

Table 2 shows the gamma pass rates obtained when using the TCM and EBT3. That is, applying a calibration curve that was generated at the same postirradiation time as the measurement film was scanned postirradiation. For five patient‐specific QA cases, the coefficient‐of‐variation (COV), defined as the standard deviation/mean across all time points postirradiation is shown in the last row. The maximum COV was found to be 3.0% across all cases with an average of 1.4% when using the TCM. A normalized plot of these data is shown in the top axes of Figure 6 “EBT 3‐ with TCM.” Table 3 shows the same patient gamma pass‐rate results, however, in this instance, a single calibration curve generated at 20 h postirradiation was used to convert each patient‐specific QA film to dose. These data show that not using a TCM can produce up to 70% variation in gamma pass rates at 1%/1 mm, if the standard single‐calibration curve, generated at 20 h postirradiation, was used to convert a measurement film scanned at 5 min postirradiation. The maximum COV in this scenario was found to be 39.3% with an average of 15.5%. These results demonstrate a large variation in gamma pass rates caused by postirradiation growth changes in ODnet not being taken into consideration. A normalized plot of this data is shown in the bottom axes of Figure 6, “EBT 3‐No TCM.”.

**TABLE 2:** EBT3 Gamma pass rates (1%/1 mm) when using the TCM for five patient‐specific QA cases with varying doses per fraction

Time (min)	EBT3‐1	EBT3‐2	EBT3‐3	EBT3‐4	EBT3‐5	Range (%)
5	98.0	87.3	89.0	97.3	99.5	12.2
10	99.5	97.9	94.5	99.2	99.9	5.4
15	99.7	98.6	97.4	99.6	100.0	2.6
20	99.8	99.5	98.3	99.7	100.0	1.7
25	99.7	99.8	99.1	99.8	100.0	0.9
30	99.8	99.9	99.5	99.9	100.0	0.5
60	99.2	100.0	99.9	100.0	100.0	0.8
120	99.7	100.0	99.9	100.0	100.0	0.3
180	99.0	100.0	100.0	100.0	100.0	1.0
240	99.9	100.0	100.0	100.0	100.0	0.1
300	99.7	100.0	100.0	100.0	100.0	0.3
600	99.9	100.0	100.0	100.0	100.0	0.1
720	99.9	100.0	100.0	100.0	100.0	0.1
840	99.9	100.0	100.0	100.0	100.0	0.1
960	99.8	100.0	100.0	100.0	100.0	0.2
1080	99.3	100.0	100.0	100.0	100.0	0.7
1200	100.0	100.0	100.0	100.0	100.0	0.0
COV (%)	0.5	3.0	2.8	0.6	0.1	–

The COV (standard deviation/mean) for each column is shown in the last row and the range (maximum‐minimum) across all five cases at time *t* is shown in the last column.

**TABLE 3:** EBT3 Gamma pass rates (1%/1 mm) when using a single calibration curve (not using the TCM) for five patient‐specific QA cases with varying doses per fraction

Time (min)	EBT3‐1	EBT3‐2	EBT3‐3	EBT3‐4	EBT3‐5	Range (%)
5	32.9	33.0	29.8	25.0	68.3	43.3
10	36.9	41.2	33.8	39.4	83.1	49.3
15	46.3	41.0	36.7	49.2	87.7	51.0
20	49.6	43.8	39.1	54.2	90.9	51.8
25	54.5	47.3	41.2	61.0	92.4	51.2
30	53.4	57.4	42.7	64.7	93.8	51.1
60	62.9	58.6	52.7	80.3	96.8	44.1
120	70.5	75.5	70.9	93.0	98.8	28.3
180	81.9	87.9	82.9	94.0	99.2	17.3
240	90.7	93.1	90.8	97.2	99.4	8.7
300	94.0	96.6	94.3	98.2	99.6	5.6
600	97.5	99.8	99.5	99.8	99.9	2.4
720	98.6	100.0	99.7	99.9	100.0	1.4
840	98.8	100.0	99.9	100.0	100.0	1.2
960	99.7	100.0	100.0	100.0	100.0	0.3
1080	99.9	100.0	100.0	100.0	100.0	0.1
1200	100.0	100.0	100.0	100.0	100.0	0.0
COV (%)	32.3	34.1	39.3	30.8	8.7	–

The COV for each column is shown in the last row and the range across all five cases at time t is shown in the last column.

**FIGURE 7:**
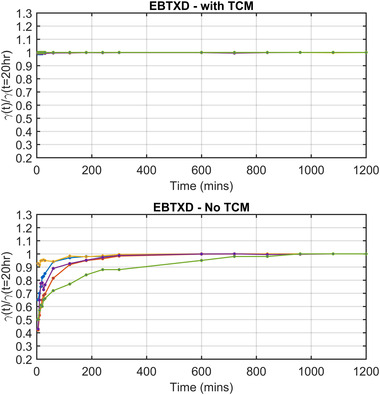
EBT‐XD normalized gamma pass‐rates at 1%/1 mm at each time point postirradiation using a TCM (top) and using a single calibration curve generated at 20 h postirradiation (bottom). Each gamma pass‐rate score is normalized to the gamma pass rate of the film at 20 h.

Table 4 shows the gamma pass rates obtained when using the TCM and EBT‐XD. Table 5 shows the same patient gamma pass‐rate results, however, in this instance, a single calibration curve generated at 20 h postirradiation was used to convert each patient‐specific QA film to dose. Table 5 shows up to 58% variation in gamma pass rates when a single calibration curve is used (EBT‐XD‐2 Case). These results again demonstrate a large variation in gamma pass rates when using EBT‐XD caused by postirradiation growth changes in ODnet not being taken into consideration. A normalized plot of this data is shown in the bottom axes of Figure 7 “EBT 3‐ No TCM.”

**TABLE 4:** EBT‐XD Gamma pass rates (1%/1 mm) when using the TCM for five patient‐specific QA cases with varying doses per fraction

Time (min)	EBT‐XD‐1	EBT‐XD‐2	EBT‐XD‐3	EBT‐XD‐4	EBT‐XD‐5	Range (%)
5	99.1	99.4	99.8	99.6	99.9	0.80
10	99.1	99.9	99.7	99.9	99.9	0.80
15	99.1	100.0	100.0	99.3	99.9	0.90
20	99.4	100.0	100.0	99.3	99.9	0.70
25	99.6	100.0	99.8	99.9	99.9	0.40
30	99.7	100.0	100.0	99.3	99.8	0.70
60	99.9	100.0	100.0	99.6	99.9	0.40
120	100.0	100.0	99.6	99.7	99.9	0.40
180	100.0	100.0	99.9	99.9	100.0	0.10
240	100.0	100.0	100.0	99.8	100.0	0.20
300	100.0	100.0	100.0	99.9	100.0	0.10
600	100.0	100.0	99.9	99.9	100.0	0.10
720	100.0	100.0	100.0	99.5	100.0	0.50
840	100.0	100.0	100.0	99.9	99.9	0.10
960	100.0	100.0	100.0	100.0	100.0	0.00
1080	100.0	100.0	99.8	100.0	100.0	0.20
1200	100.0	100.0	100.0	100.0	100.0	0.00
COV (%)	0.4	0.1	0.1	0.3	0.1	–

The COV for each column is shown in the last row and the range across all five cases at time t is shown in the last column.

**TABLE 5:** EBT‐XD Gamma pass rates (1%/1 mm) when using a single calibration curve (not using the TCM) for five patient‐specific QA cases with varying doses per fraction

Time (min)	EBT‐XD‐1	EBT‐XD‐2	EBT‐XD‐3	EBT‐XD‐4	EBT‐XD‐5	Range (%)
5	65.0	42.0	92.7	42.8	50.3	50.7
10	70.0	53.3	91.6	64.9	57.5	38.3
15	75.0	59.7	94.7	77.5	61.0	35.0
20	82.0	64.9	95.1	78.0	60.3	34.8
25	83.3	68.3	95.4	72.7	65.0	30.4
30	84.6	69.4	94.9	76.4	66.3	28.6
60	93.8	81.5	94.2	88.9	71.9	22.3
120	97.1	91.8	98.3	92.6	76.7	21.6
180	97.9	94.9	97.8	95.2	84.2	13.7
240	98.0	96.3	98.5	97.3	88.0	10.5
300	98.6	98.3	99.5	98.8	88.5	11.0
600	99.9	99.8	100.0	99.9	94.6	5.4
720	100.0	99.9	100.0	100.0	97.8	2.2
840	100.0	99.9	100.0	99.5	97.7	2.3
960	100.0	100.0	100.0	99.7	100.0	0.3
1080	100.0	100.0	100.0	100.0	100.0	0.0
1200	100.0	100.0	100.0	100.0	100.0	0.0
COV (%)	12.6	22.8	2.9	18.1	21.1	–

The COV for each column is shown in the last row and the range across all five cases at time *t* is shown in the last column.

**FIGURE 8:**
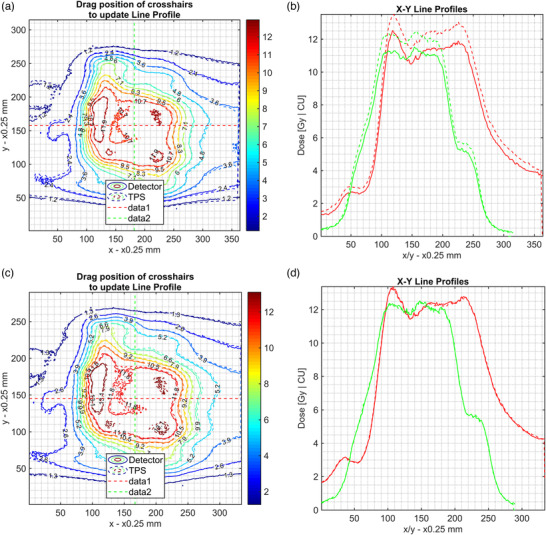
Demonstration of the effect of using a calibration curve that was created at a different time postirradiation for a patient‐specific QA spine case. (a) Shows the registered isodose overlay between the film (solid line) and TPS dose plane (dashed line) and the location of a line profile. (b) Shows the horizontal and vertical dose profiles for the film and TPS planes, respectively, where a 24‐h calibration curve was applied to a measurement film read out at 1 h postirradiation. (c) and (d) Show the same data but with a TCM applied and a correct calibration curve assigned

As a visual example of this effect, Figure 8 shows a spine SBRT patient‐specific QA result without (a–b) and with a TCM (c–d). Applying a calibration curve to a measurement film that was scanned at a shorter postirradiation time, results in an offset across the distribution that does not reflect the reality of what was planned by the TPS and delivered by the linear accelerator.

### 3.4 Clinical patient‐specific QA implementation and initial results

The results of the initial testing of this method in clinical practice across two planning systems and linear accelerators are shown in Table 6. The average difference from the actual patient‐specific QA results for these cases using the TCM was found to be 2.8%, with a range of 5.3%, and a maximum difference of 5.5%, at arbitrary time points postirradiation. When a single calibration curve generated at 24 h (Cases 1 and 2) or 12 h (Cases 3–6) was used at these time points, the average difference from the actual result was found to be 11.1% with a range of 47.9%.

**TABLE 6:** Results of the patient‐specific QA trials at two facilities

Patient‐specific QA Case #	Postirradiation time (minutes)	TCM (2%/1 mm)	Single calibration curve (2%/1 mm)	"True" Result (2%/1 mm)	True–TCM (%)	True–Single calibration (%)
Case 1	8	89.0	99.4	87.9	1.1	11.5
	18	83.9	99.3		4.0	11.4
	28	84.2	98.7		3.7	10.8
	58	85.2	97.8		2.7	9.8
	298	84.2	90.0		3.7	2.1
	598	84.0	87.9		4.0	0.0
	1198	88.0	88.0		0.1	0.1
Case 2	12	91.2	94.3	91.0	0.3	3.3
	22	89.8	96.6		1.1	5.6
	28	89.2	97.2		1.8	6.3
	58	89.6	96.9		1.4	5.9
	297	85.5	91.9		5.5	0.9
	597	89.7	91.9		1.3	1.0
	1197	91.7	91.0		0.7	0.0
Case 3 PTV 1	40	74.9	54.7	80.3	5.4	25.7
Case 3 PTV 2	30	96.7	86.8	97.8	1.1	10.9
Case 4 PTV 3	20	89.9	47.6	95.5	5.5	47.9
Case 5 PTV 2	30	97.5	87.0	99.1	1.6	12.1
Case 5 PTV 4	20	99.2	82.2	98.1	1.1	15.9
Case 6 PTV 2	40	90.2	77.3	94.9	4.7	17.6
Case 6 PTV 3	50	96.7	80.7	92.9	3.8	12.2

Patient cases were scanned at times chosen by each participant and compared to the “True” result which is the result obtained through each facility's set methodology and usual scan postirradiation time.

## 4 DISCUSSION

The aim of this work was twofold. (1) To investigate and present a comparison between EBT3 and EBT‐XD in terms of postirradiation coloration changes. (2) To create an automated workflow to allow radiochromic film (EBT3/XD) to be scanned and converted to dose accurately at any postirradiation time, thus, negating the effects investigated in Aim 1. In this work, we have provided validation of an automated workflow to create a TCM. The TCM is defined as a series of calibration curves distinct in scan postirradiation time, that is automatically generated both in the scanning and processing phases. We have demonstrated that this method can be used to automatically account for postirradiation OD growth in clinical patient SRT/SBRT cases.

We have also compared the postirradiation characteristics of EBT3 and EBT‐XD in terms of OD growth. It is well established that radiochromic film undergoes continual postirradiation changes in OD for a period of time, with the change in OD in the EBT model series (EBT, EBT2, EBT3, and EBT‐XD) and MD‐V3, HD‐V2 being proportional to the log of time after irradiation.^14^, ^25^ The recent publication of AAPM Task Group 235′s report on radiochromic film dosimetry ^20^ states that after 24 h when the polymerization is nearly complete and the rate of change in OD is low. We have shown this to be true with a comprehensive evaluation of both EBT3 and EBT‐XD film, with dose levels from 0 to 18 Gy. This work also demonstrates that these effects can be overcome after as little as 5 min post‐irradiation by applying a calibration curve that was scanned at, or as close as possible to the time the measurement film was scanned.

In this work, it is important to note that we did not consider the time taken for irradiation of each calibrate, that is, starting the stopwatch only after the final calibrate was exposed, then beginning scanning 5 min later. For ten calibrates, the time taken for exposure was approximately 15–20 min with a 6 FFF beam, utilizing a 1200 MU/min dose rate. The potential additional difference in time between a calibration set irradiation and a particular measurement irradiation was not found to have a significant impact as shown in the results for the patient cases in section 3.2. However, it is still advisable to wait 30 min so that any timing error is reduced. This is evident from Tables 2 and  4 where spurious results at 5 min postirradiation for EBT3‐2 and EBT3‐3 can be seen, which then approach the ground‐truth pass rate after 30 min. In terms of an efficiency gain, there is little difference in a clinical setting between getting results at 5 min postexposure and waiting an additional 25 min.

This work also highlights in detail the importance of minimizing the time difference between the calibration curve scan and measurement scan postirradiation. Large differences in gamma results can be seen when using a single calibration curve generated at 20 h postirradiation, to convert measurement films to dose, that were scanned with a shorter time delay postirradiation. These differences increase with increasing difference in time between the postirradiation scan time of the calibration set and measurement scan. Examining Figures 6 and 7, we can see that after approximately 600 min (10 h), gamma pass rates (1%/1 mm) are within 2.5 and 0.2% of those at 20 hs postirradiation for EBT3 and EBT‐XD cases respectively, with the exception being EBT‐XD‐5 which is within 5.4%.

There are two purported solutions to correct this post‐irradiation OD growth reported in TG‐235.^20^, ^32^ The most well known is the “One‐Scan” protocol developed by Lewis et al.^29^ This method provides a way to account for postirradiation OD growth by a linear scaling of the response curves based on two (or more) reference films of known dose. Their work also demonstrated the accuracy of this method in accounting for postirradiation OD growth. However, the dose ranges of their validation for IMRT and VMAT patient cases were only ∼2 and ∼1.2 Gy respectively (taken from Figure 10 of Ref. [29]). As we have shown in Figures 3 and 5, the postirradiation growth in OD_net_ is also a function of dose, and while the method outlined in ^29^ may be suitable for small dose ranges, the greater the range of doses in the measurement film, such as the case with common stereotactic clinical cases, could see greater uncertainty when using only two dose‐recalibration points. What is needed for accurate conversion to dose at any postirradiation time is a full calibration curve covering the full range of doses that were also generated at the same postirradiation time of the measurement film being scanned. Such a method is detailed in this work. The use of the “One‐Scan” protocol in conjunction with this method could potentially improve the accuracy of the TCM method further as it includes a correction of the scanner response in the linear scaling of the measurement film.

In a clinical implementation trial, we have shown that we can obtain patient‐specific QA results on average within 3% (for gamma criteria of 2%/1 mm) of the true result at a range of times postirradiation (Table 6). Interestingly and worrisome is the clinical patient‐specific QA implementation and initial results for Cases 1 and 2 that show a false‐positive result if a 24‐h calibration curve is used on a patient measurement scan that is read out within an hour postirradiation. Cases 3–6 may have shown this to be true, also they had been scanned at a similarly short postirradiation time; however, these cases were read out at longer times postirradiation, 20 –50 min, respectively.

We have demonstrated the application of a TCM with a high temporal resolution of 5‐min intervals postirradiation. It is important to note, however, that any arbitrary temporal resolution to fit the clinic's needs could be derived. In clinical use, a 30‐min interval over 24 h for the scan time would be sufficient and would result in fewer images produced (48 instead of 288 images and subsequent calibration curves). Or one may choose to generate two TCMs, one at 5‐min intervals, for the first‐hour postirradiation and every hour thereafter. If there is a time point in the TCM that corresponds to the postirradiation time when the measurement film was scanned, the uncertainty will be minimized. This method can also be applied with currently available software although it would be more laborious as no software currently automates the process. The AutoHotKey software, however, could be implemented anywhere and be used to scan the calibration films at fixed intervals automatically at the very least.

## 5 CONCLUSIONS

We have presented a method for creating a TCM to allow radiochromic film to be processed quickly and accurately postirradiation. This method significantly reduces the waiting periods that are well known to hinder the immediate use of radiochromic film in clinical use cases due to the postirradiation growth in OD. All steps, including scanning, processing images, and creating calibration curves, as well as application of the TCM to measurement cases, are automated. The eFilmQA software only requires the user to enter the time interval between repeat calibration scans when generating the TCM and the time interval posirradiation that the measurement film was scanned to apply the correct TCM.
